# Protective effects of a Modified Vaccinia Ankara-based vaccine candidate against Crimean-Congo Haemorrhagic Fever virus require both cellular and humoral responses

**DOI:** 10.1371/journal.pone.0156637

**Published:** 2016-06-07

**Authors:** Stuart D. Dowall, Victoria A. Graham, Emma Rayner, Laura Hunter, Robert Watson, Irene Taylor, Antony Rule, Miles W. Carroll, Roger Hewson

**Affiliations:** Public Health England, Porton Down, Salisbury, Wiltshire, United Kingdom; University of Liverpool, UNITED KINGDOM

## Abstract

Crimean-Congo Haemorrhagic Fever (CCHF) is a severe tick-borne disease, endemic in many countries in Africa, the Middle East, Eastern Europe and Asia. There is no approved vaccine currently available against CCHF. The most promising candidate, which has previously been shown to confer protection in the small animal model, is a modified Vaccinia Ankara virus vector expressing the CCHF viral glycoprotein (MVA-GP). It has been shown that MVA-GP induces both humoral and cellular immunogenicity. In the present study, sera and T-lymphocytes were passively and adoptively transferred into recipient mice prior to challenge with CCHF virus. Results demonstrated that mediators from both arms of the immune system were required to demonstrate protective effects against lethal challenge.

## Introduction

Crimean-Congo haemorrhagic fever (CCHF) is an acute tick-borne zoonotic disease. The causative agent, CCHF virus (CCHFv), has the most extensive geographical distribution of the medically important tick-borne viral diseases [1] with a distribution over much of Asia, the Middle East, Africa and expanding areas of south-eastern Europe [2]. The continued spread of the *Hyalomma* tick vector through climate change and modern farming practices has resulted in the virus becoming established in territories in which it was not previously endemic; its introduction to Turkey, Greece and Spain being testament to this [3].

Recognised antiviral compounds (e.g. ribavirin) or vaccines have not proved to be effective against CCHFv in controlled trials [4, 5]. The only currently available vaccine is that produced in Bulgaria, which is made in suckling mouse brain, inactivated by chloroform, heated at 58°C and adsorbed on aluminium hydroxide [6]. Although it has been shown to elicit immunity [7], there is currently no evidence directly demonstrating efficacy. It is unlikely that due to its crude preparation it will ever gain widespread international regulatory approval.

Recent vaccine approaches for CCHF include a DNA vaccine expressing the entire open reading frame of the M segment which includes the envelope glycoproteins of CCHFv and has been shown to elicit antibody responses [8]. Similarly, another CCHF vaccine candidate based on transgenic plants expressing the CCHFv glycoprotein has been shown to induce antibody [9]. However, neither of these vaccines has been tested in an animal model. Work on CCHFv was hampered by a lack of a suitable animal model, until 2010, when mice deficient in STAT-1 [10] or type-I interferon receptor (A129, IFN-α/βR^-/-^) [11] were demonstrated to be susceptible to infection. The STAT-1 knockout mice exhibited selective signalling defects in their response to all three types of IFNs (type I, IFN-α and –β; type II, IFN-γ; and type III, IFN-λ) which leads to a complete abolishment of the intracellular IFN response [12], and therefore the A129 mice offer the more robust and intact immune system of the two models. A129 mice show no overt anomalies, but are unable to control with certain viral infections, despite otherwise normal immune responses [13, 14]. Consequently, they provide a useful model for investigating the adaptive immune response and performing active protective studies under stringent, frequently lethal, conditions [15].

To date, only two CCHF vaccine candidates have been reported to confer protection against lethal challenge in the A129 mouse model. One used a cell culture based vaccine, which required the growth of live virus followed by an inactivation procedure (which included treatment with formaldehyde) and showed partial protection [16]. Due to the high biological containment needed to handle live CCHFv, this approach is unlikely to be applicable to large scale vaccine manufacture and suffers from the same limitations as the Bulgarian vaccine approach in being unlikely to gain international regulatory approval. The second vaccine showed complete protection against CCHFv using a modified vaccinia Ankara (MVA) virus vector expressing the CCHFv glycoprotein (MVA-GP) [17]. Therefore, it has been demonstrated that despite lacking the type-I interferon receptor, A129 mice maintain sufficient adaptive immunity to allow protection from a lethal challenge dose of CCHFv. The A129 mouse strain has also been used to decipher the protective role of antibodies induced by a novel vaccine candidate in protection against another arbovirus, Chikungunya virus [18]. Therefore, its application to the study of vaccination responses is well documented.

Since the MVA-GP vaccine induced both antibodies and T-cells to the CCHFv antigen, the relative contribution of each in eliciting protective immunity is unknown. Therefore, this study was designed to ascertain whether immune sera, CD3^+^ T-lymphocytes, or both were required to exert protective effects.

## Materials and Methods

### Cells

BHK-21 cells (American Type Culture Collection, USA) were cultured in modified essential eagle medium (Sigma, UK) supplemented with 10% foetal bovine serum, 2 mM L-glutamine, 100 U penicillin and 0.1 mg/ml streptomycin (Sigma). Chick Embryonic Fibroblast (CEF) cells (Institute for Animal Health, UK) were cultured in Dulbecco’s modified eagle medium (Sigma) supplemented as above. SW13 and VeroE6 cells (European Collection of Cell Cultures, UK) were maintained in Leibovitz’s L-15 medium containing Glutamax (Life Technologies, UK) supplemented with 10% foetal bovine serum (Sigma).

### Viruses

MVA strain 1974/NIH clone 1 (kindly supplied by Prof B. Moss, NIH) was used for the vaccine construct. The recombinant MVA-GP vaccine was prepared as described previously [17]. Virus titre was determined by plaque assay in BHK-21 cells. CCHF virus strain IbAr10200 was initially prepared from suckling mouse brain homogenate and then passaged twice in SW13 cells (European Collection of Cell Cultures, UK). Viral titre was determined by focus-forming unit (ffu) assay in Vero cells.

### Ethics statement

All procedures were undertaken according to the United Kingdom Animals (Scientific Procedures) Act 1986. These studies were approved by the ethical review process of Public Health England, Porton Down, UK and the Home Office, UK via an Establishment Licence (PEL PCD 70/1707) and project licences (30/2476 and 30/2697).

### Animals

Female A129 (IFN-α/βR^-/-^) mice aged 5–8 weeks were sourced from an approved supplier (B&K Universal, UK). Mice were housed in negative pressure flexible isolators to provide protection from opportunistic infections in a Containment Level 4 facility. Food and water were available *ad libitum*. All efforts were made to minimise animal suffering; manipulations were minimised and endpoints were limited to a moderate severity rating. For culling at Containment Level 4, mice were anesthetised with isofluorane gas and then a cervical dislocation was performed.

### Immunisation of mice

Mice were injected into the caudal aspect of the proximal hind limb musculature with 10^7^ plaque-forming units (pfu) per animal of MVA-GP (n = 45 mice) or MVA-1974 (n = 18 mice) diluted in endotoxin-free PBS. A total volume of 100 μl was delivered equally across two sites. Animals received a booster vaccination 14 days later. Control animals received 10^7^ pfu of non-recombinant MVA 1974 or an equivalent volume of saline. Twelve days after the booster vaccination, a tail bleed was collected from animals. On day 13 post-booster vaccination, a random selection of animals were euthanised from the MVA-GP (n = 36) and MVA-1974 (n = 9) groups with blood collected into serum separation tubes (Becton Dickinson, UK) and spleens collected into RPMI 1640 media (Sigma, UK) from all euthanised animals.

### Passive transfer with immune sera

Blood from vaccinated animals was left to fully clot before centrifuging at 1300 RCF for 10 minutes. Sera collected the day before euthanasia were used in ELISA assays against the CCHFv Gn protein, as previously described [17], in order to confirm successful vaccination of the animals. Blood collected at the terminal bleed was used for passive transfer into recipient animals, with sera from the individual animals being pooled and then 200 μl being delivered via the intraperitoneal (i.p.) route. The passive transfer procedure was conducted within 6 hours of the blood being collected from the donor animals.

### Adoptive transfer with immune CD3^+^ T lymphocytes

Spleens from immunised animals had any excess fat removed before being pooled according to the immunisation regime and dissociated in a C-tube using a GentleMACS (Milteny Biotech, UK). Splenocytes were filtered with a 70 μm filter and washed with RPMI 1640 medium containing 2% foetal bovine serum before being treated with 0.83% ammonium chloride solution for 5 minutes to lyse erythrocytes. Finally, they were washed with sterile PBS (Invitrogen, UK). For all procedures, cells were kept on wet ice and reagents were pre-cooled. CD3^+^ T cell enrichment was performed using negative selection with a commercially-available procedure (STEMCELL Technologies, UK). Briefly, splenocytes were resuspended in robosep buffer at a concentration of 10^8^ cells/ml before the addition of rat serum (to reduce non-specific binding) and a CD3^+^ antibody cocktail. Tubes were mixed for 10 minutes before rapid spheres were added and incubated for 3 minutes. The tubes were then placed on a magnet and the unbound fraction was removed, containing the enriched CD3^+^ T cells. Cells were counted and diluted with sterile PBS to give a concentration of 5x10^7^ cells/ml. For adoptive transfer studies, 200 μl of cell suspension was intraperitoneally delivered, equating to 1x10^7^ cells. The adoptive transfer procedure was conducted within 6 hours of the spleens being collected from the donor animals. To confirm CD3^+^ enrichment, cells before and after the selection process were assessed via flow cytometry after staining with a CD3-FITC antibody (Becton Dickinson, UK) and using an anti-rat IgG2-FITC isotype antibody (Becton Dickinson, UK) to control for non-specific staining. The data were collected on an FC500 flow cytometer (Beckman Coulter) and analysed with CXP analysis version 2.1 software (Applied Cytometry Systems).

### Challenge with CCHFv

One day after the administration of sera, CD3^+^ T-lymphocytes, or both, mice (n = 9 per group) received 200 ffu CCHF virus strain IbAr10200 intradermally in the midline of the lumbar region in a volume of 100 μl divided equally across two sites. 50 μl is the maximum recommended volume for intradermal inoculation of mice [19] and confirmation of intradermal delivery was seen by a visible bleb formation under the skin. Animals immunised with MVA-1974 and MVA-GP (n = 9 per group) were simultaneously challenged. Post-challenge, animals were weighed and body temperature measured daily by a subcutaneously located temperature chip. In addition, they were observed for clinical signs of disease twice daily (arching, ruffled fur, lethargy and immobility). Criteria for euthanasia on welfare grounds consisted of 20% weight loss or observation of two abnormal clinical signs. At 4 days post-challenge, randomly selected animals were euthanised and samples of blood, spleen and liver were collected for viral load studies. Spleen and liver samples were also collected for histopathological examination.

### Viral load determination

Whole blood (100 μl) was collected into RNA Protect Animal Blood tubes (Qiagen) and stored at -80°C. Tubes were thawed, inverted and left for a further 2 hours at room temperature to ensure efficient cell lysis. Samples were treated with Red Blood Cell Lysis Solution (Miltenyi Biotec) before purification of total RNA using an RNeasy Mini kit (Qiagen).

For viral load analysis, spleen and liver samples were collected into RNALater (Qiagen) and stored at -80°C. Thawed tissue was transferred to RLT buffer (Qiagen), homogenised by passing through a 70 μm sieve and then treated using an RNeasy Mini kit (Qiagen) for extraction of total RNA.

CCHFv S segment was detected by RT-PCR on the ABi 7500 RT-PCR platform as previously described [20], with cycling conditions adjusted to those described in the QuantiFast probe assay: 50°C for 20 min and 96°C for 5 min, followed by 45 cycles of 95°C for 15 sec and 60°C for 30 sec (with quantification analysis of fluorescence performed at the end of each 60°C step), and final cooling of 40°C for 30 sec. A synthetic S segment of known concentration was used to quantify S segment copy number in blood and tissue samples. All reactions were run in triplicate.

To normalise the CCHFv expression data, the hypoxanthine guanine phosphoribosyl transferase (HPRT) housekeeping gene was used. A one-step RT-PCR with singleplex detection was performed targeting an 89 bp product in the mouse HPRT gene (NCBI Reference sequence NM_013556) using the QuantiFast probe assay (Qiagen) and the ABi 7500 RT-PCR platform. CT values for CCHFv and HPRT were each inverted by subtracting the CT value from 45 (the total number of cycles), where CT is the number of cycles to reach the fluorescence threshold value. The mean value of CCHFv was then divided by the mean value of the HPRT reference gene for each sample.

### Histological analysis

Samples of spleen and liver were placed in 10% neutral buffered formalin for 7 days and processed routinely to paraffin wax. Sections were cut at 3–5 μm, stained with haematoxylin and eosin (H&E) and examined microscopically. Lesions referable to infection with CCHF virus were scored subjectively using the following scale: normal, minimal, mild, moderate and marked.

For immunohistochemistry (IHC), formalin-fixed, paraffin-embedded sections of spleen and liver, cut between 3–5 μm, were mounted on positively charged X-tra Adhesive slides (Leica Biosystems, UK), deparaffinised and rehydrated. Immunohistochemical staining was achieved using a BOND-MAX Immunostainer (Leica Microsystems, UK) and a Novacastra Bond Intense R (Leica Biosystems) detection kit. A heat-induced epitope retrieval cycle with buffer ER1 (Leica Biosystems) was performed for 20 minutes. Slides were incubated with rabbit serum (4%) (Abcam, Cambridge, UK) for 20 minutes before avidin/biotin blocking (15 minutes each) (Abcam). Polyclonal antibody raised in sheep immunised against recombinant CCHFv nucleoprotein (kindly provided by Dr John Barr, University of Leeds, UK) was incubated with the tissue for 30 minutes, and this was followed by incubation with a biotinylated rabbit anti-sheep polyclonal antibody (Abcam) at a dilution of 1:500, for 10 minutes, resulting in a brown stain. Haematoxylin was used as the counterstain. Positive and negative control slides were included. Immunolabelled slides were evaluated using light microscopy.

## Results

### Preparation of sera and enriched CD3^+^ T cells

**Fig 1:**
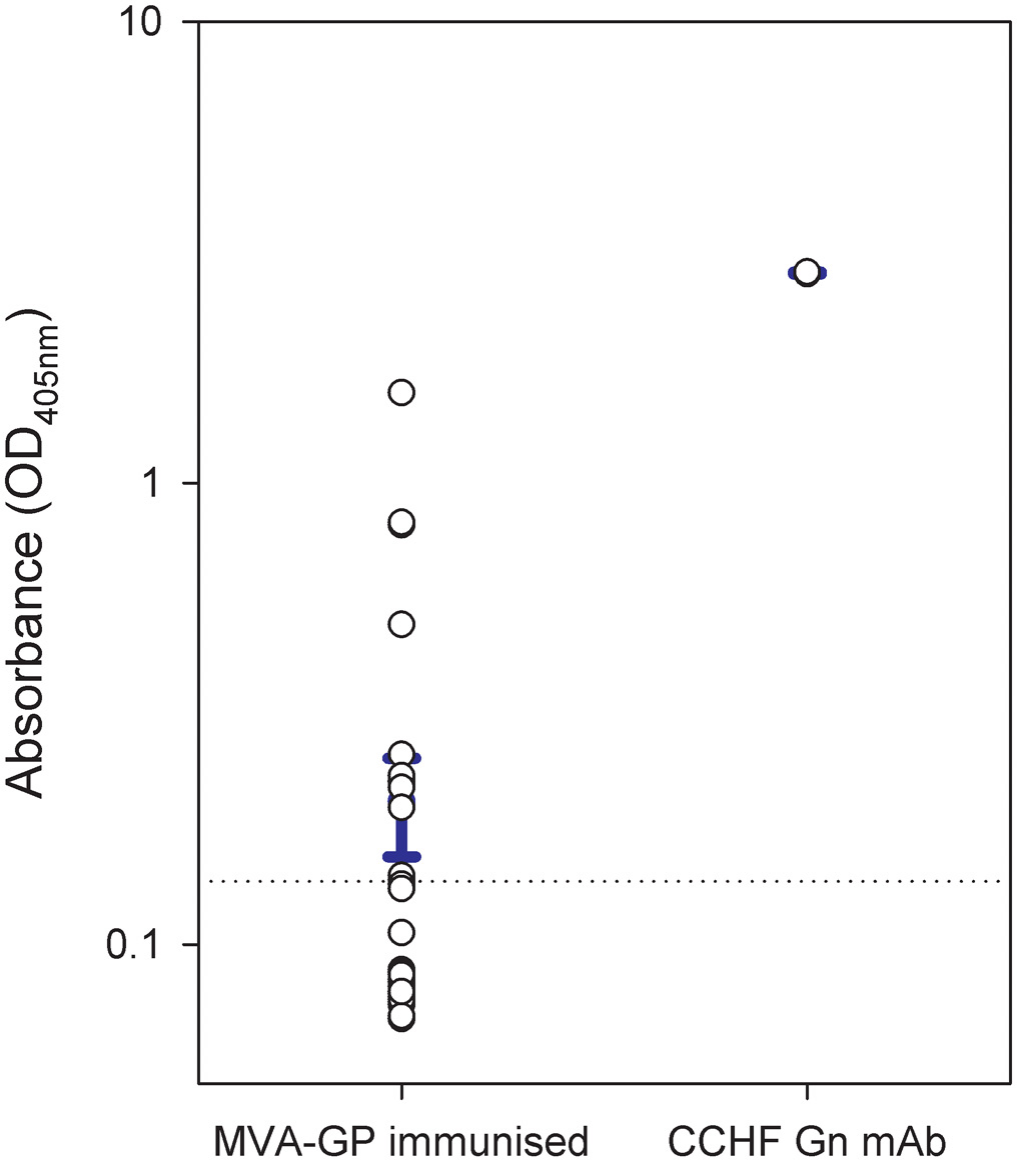
Measurement of antibody binding to CCHF virus Gn protein from mice immunised with MVA-GP. The absorbance readings from an ELISA assay using the Gn protein as antigen were plotted from all individual donor animals (n = 36). A control antibody preparation from a mouse hybridoma cell line developed against the Gn protein was used as a positive control. The error bar denotes the mean and standard error. The dotted line represents the cut-off level based on mean absorbance + 2 standard deviations of unvaccinated guinea pig control sera.

On the day before immunised animals were scheduled to be culled, blood samples from individual animals were collected and analysed for antigen-specific binding by ELISA. Samples from immunised mice demonstrated binding to the CCHF Gn protein at varying levels, with some animals demonstrating responses (n = 12) whilst others showed no specific binding (n = 24) (Fig 1). Upon culling of animals, sera were pooled from all similarly immunised animals to produce a population-based pool prior to being used for passive transfer.

During preparation of splenocytes from immunised animals, samples were labelled with fluorescently-conjugated antibodies specific to lymphocyte markers and analysed by flow cytometry before and post-enrichment. The percentage of CD3^+^ T-lymphocytes in the splenocytes of MVA-1974 and MVA-GP immunised animals were 25.5% and 32%, respectively. After enrichment of CD3^+^ cells in the splenocytes of MVA-GP immunised animals the contribution increased to 72.4%. The viability of all cell preparations was >95%.

### Survival

**Fig 2:**
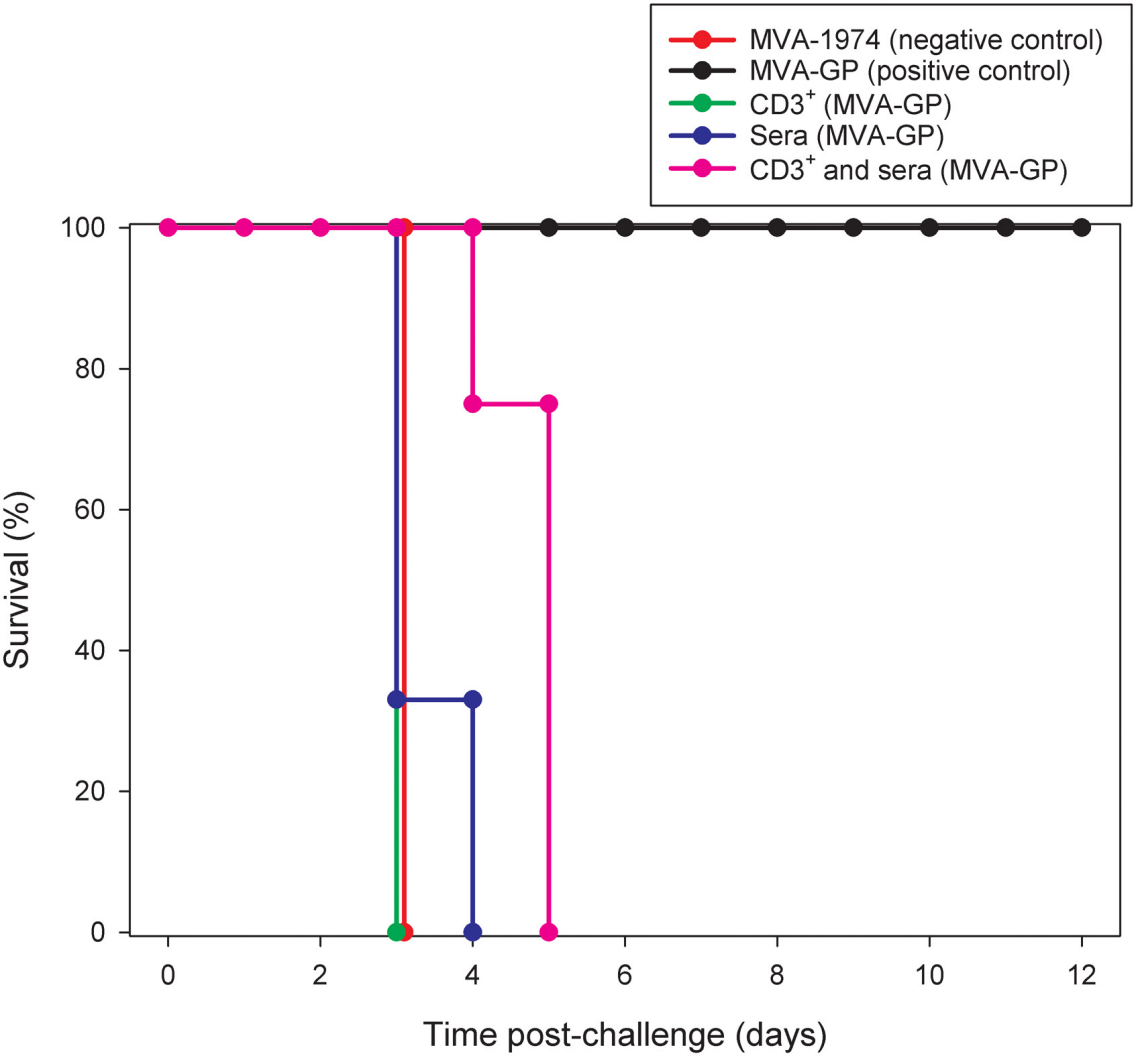
Survival of A129 mice vaccinated with MVA-1974, MVA-GP or which received sera, CD3^+^ T cells, or both from MVA-GP immunised animals after challenge with CCHF virus. Animals were challenged with 200 ffu of CCHF virus 14 days after the booster vaccination or 1 day post-transfer of sera, CD3^+^ T cells, or both. Six animals from each group were used to assess survival post-challenge.

Mice given MVA-1974 all met humane clinical endpoints at day 3 post-challenge, whereas those which received the MVA-GP vaccine survived until the scheduled end of the study 12 days post-challenge (Fig 2). Transfer of CD3^+^ T cells or sera from MVA-GP immunised animals failed to confer any significant effects on the survival of MVA-1974 immunised animals (P = 1.000 and P = 0.197, respectively, Log-Rank survival analysis), with mean times to death of 3 and 3.33, respectively. However, transfer of both CD3^+^ T cells and sera from MVA-GP immunised animals did result in a significant increase in survival time (P = 0.005, Log-Rank survival analysis), with the mean time to death being 4.75 days post-challenge.

### Clinical signs

**Fig 3:**
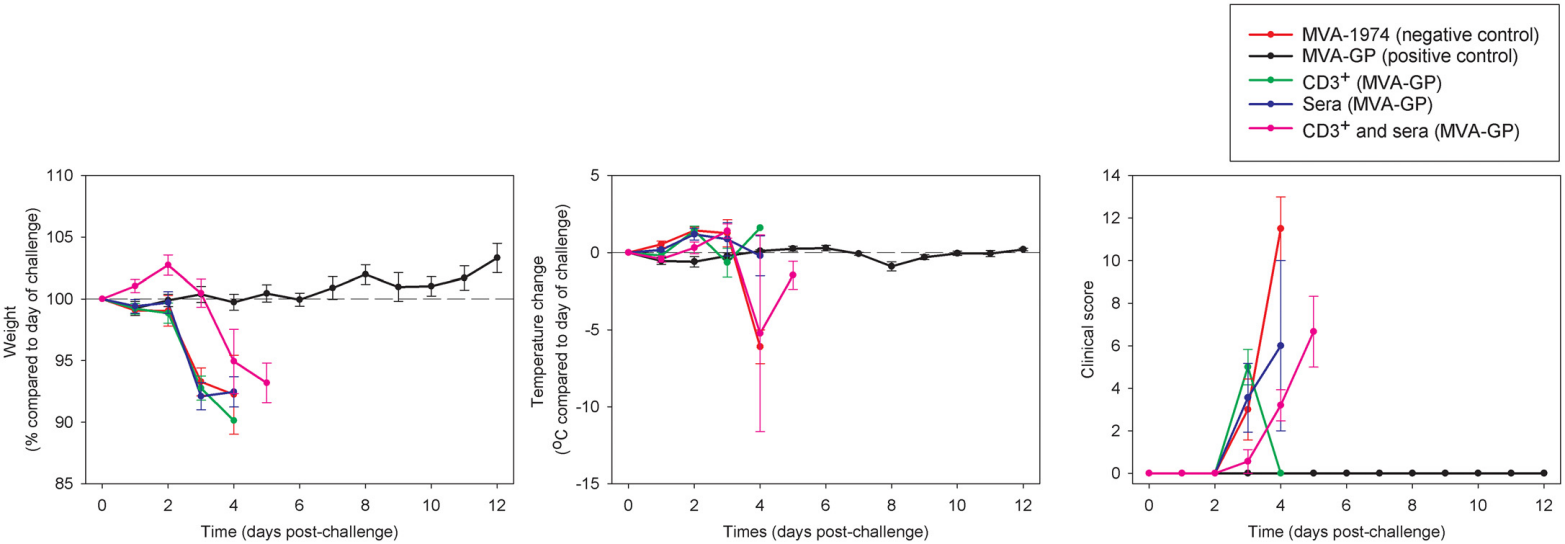
Weight changes, temperature changes and clinical signs of A129 mice vaccinated with MVA-1974, MVA-GP or which received sera, CD3^+^ T cells, or both from MVA-GP immunised animals after challenge with CCHF virus. (A) Weight changes were assessed as percentage change compared to the day of challenge. (B) Temperature changes were analysed as the °C difference compared to the day of challenge. (C) Clinical score was a numerical value based on signs recorded each morning of the study. Symbols show the mean value from all surviving animals from each group alive at that time (n = 6 per group challenged with CCHF virus) and error bars denote the standard error.

Weight changes in animals receiving CD3^+^ T cells or sera from MVA-GP immunised donors followed a similar downward trend to that observed in animals immunised with MVA-1974 (Fig 3A). The group of mice which received both CD3^+^ T cells and sera from MVA-GP immunised animals exhibited the same kinetics of weight loss as the animals just given either CD3^+^ T cell or sera, but effects were delayed by 1–2 days. Animals protected from the CCHF virus challenge after vaccination with MVA-GP did not lose weight.

Average temperature increases of 1–2°C were recorded in all groups apart from those immunised with MVA-GP (Fig 3B). Upon reaching humane clinical endpoints, several animals had a substantial fall in temperature. The group which received MVA-GP showed stable temperatures throughout the course of the study.

Numerical values were assigned to the recorded clinical signs to allow the data to be graphically represented. All groups showed clinical signs of illness apart from those immunised with MVA-GP (Fig 3C). Signs first started appearing 3 days post-challenge. However, in the animals which received both CD3^+^ T cells and sera from MVA-GP immunised animals, there was a delay before similar levels of signs appeared compared to the groups that received only CD3^+^ T cells or sera.

### Virology

**Fig 4:**
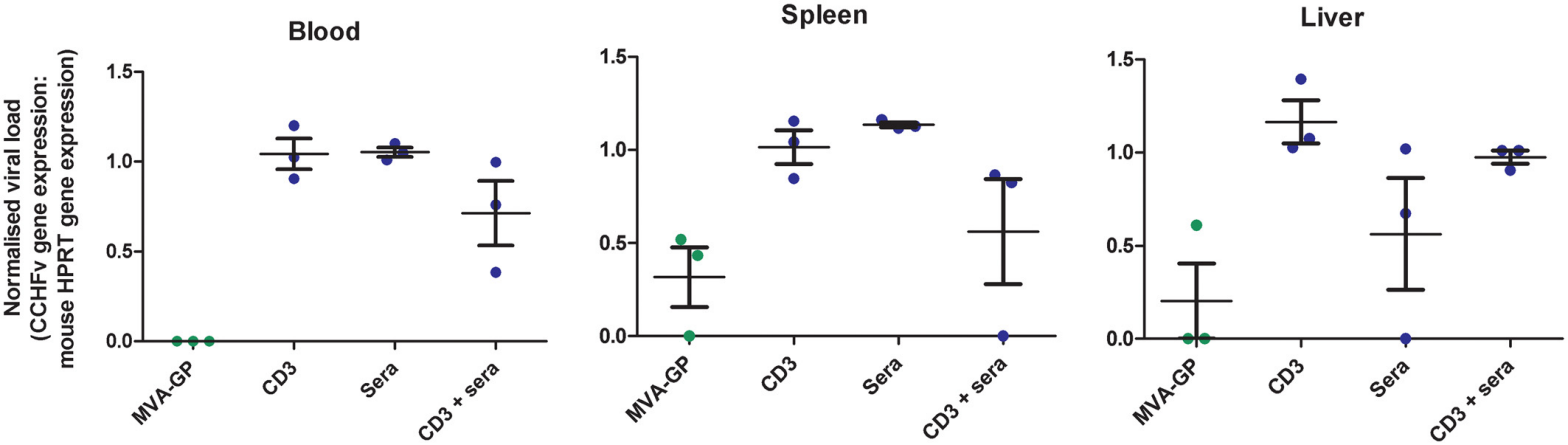
Normalised viral load analysis of CCHFv RNA by RT-PCR. A129 mice vaccinated with MVA-1974, MVA-GP or which received sera, CD3^+^ T cells, or both from MVA-GP immunised animals were challenged with CCHFv either 14 days after the booster vaccination or 1 day after transfer of immune mediators. Four days post-challenge, three randomly selected animals from each group were killed humanely and analysed by RT-PCR for levels of CCHFv gene expression (normalised to mouse HPRT gene expression). Each point represents the mean value of triplicate measurements in an individual animal. Lines show mean values and error bars denote standard error.

Samples of blood, spleen and liver were collected from 3 animals from each group 3 days post-challenge. The levels of CCHFv RNA were compared to those of the HPRT gene in order to standardise the ratio of viral RNA to a cellular house-keeping gene. Results demonstrated that in the blood, spleen and liver there were lower levels of viral RNA in the MVA-GP immunised animals then in the groups which received sera, CD3^+^ T-cells, or both (Fig 4). In the blood and spleen, there were indications that the viral RNA levels were lower in the group which received both humoral and cellular mediators. However, for the liver the responses were less distinct. Due to the small number of animals culled at this timepoint, statistical analysis was not carried out.

### Histology

**Fig 5:**
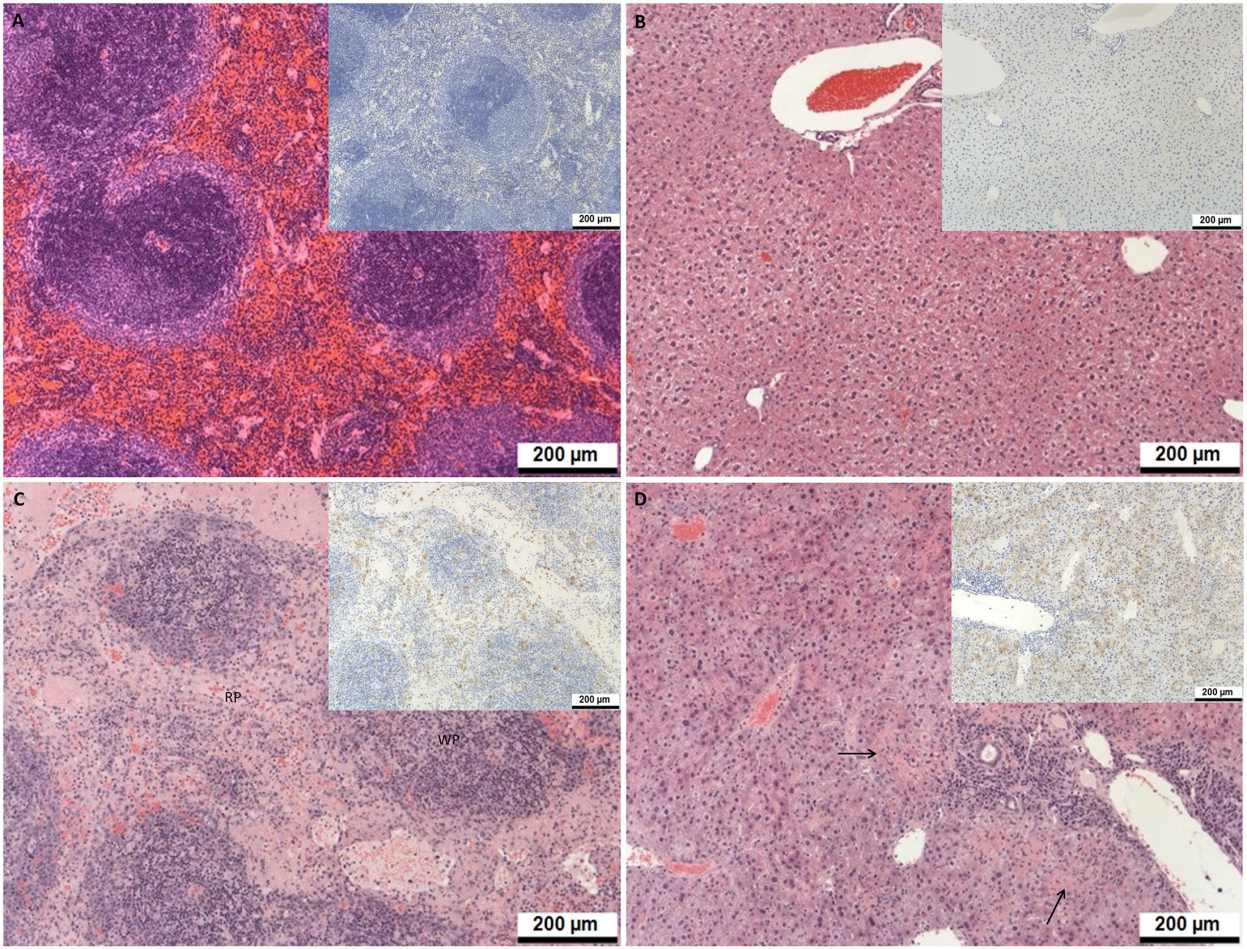
Pathological findings in the spleen (A and C) and liver (B and D) of mice post-challenge with CCHFv. (A) Spleen from mouse which received MVA-GP vaccine showing normal pathology. (B) Liver from mouse which received MVA-GP vaccine showing normal pathology. (C) Spleen from mouse which received serum from MVA-GP immunised mice with a paucity of lymphocytes in the white pulp (WP) and an increase in macrophages in the red pulp (RP). Numerous, scattered cells stained positive for CCHFv antigen. (D) Liver from mouse which received serum from MVA-GP immunised mice showing multifocal areas of hepatocyte necrosis (arrows) with inflammatory cell infiltration. Numerous, scattered cells stained positive for CCHFv antigen. Main image, HE staining; inset image, IHC staining.

To provide a snapshot of responses in the spleen and liver of challenged mice, tissues collected from 3 animals per group 3 days post-challenge were analysed for histological changes and the presence of viral antigen by immunohistochemistry. Results showed that the MVA-GP immunised animals had neither evidence of lesions associated with CCHFv infection nor the presence of viral antigen, whereas those immunised with empty MVA or receiving sera, CD3^+^ T cells, or both from immunised animals had both lesions detected and viral antigen present (Table 1 and Fig 5). Of the six samples in the group that received both CD3^+^ T cells and sera, lesion severity was scored mild in five and moderate in 1 of the liver samples. This indicates that the lesions were less severe in this group at this timepoint.

**Table 1:** Severity of microscopic lesions and presence of viral antigen in tissues from vaccinated and recipient mice 3 days post-challenge with CCHFv.

Organ	Description	MVA-1974 immunised			MVA-GP immunised			CD3^+^ (MVA-GP)			Sera (MVA-GP)			CD3^+^ & sera (MVA-GP)		
		M1	M2	M3	M4	M5	M6	M7	M8	M9	M10	M11	M12	M13	M14	M15
Spleen	Severity of acute lesions	Mod	Mild	Mod	WNL	WNL	WNL	Mkd	Mod	Mild	Mkd	Mkd	Mod	Mild	Mild	Mild
	Presence of viral antigen	Yes	Yes	Yes	No	No	No	Yes	Yes	Yes	Yes	Yes	Yes	Yes	Yes	Yes
Liver	Severity of acute lesions	Mod	Mild	Mod	WNL	WNL	WNL	Mod	Mild	Mod	Mkd	Mod	Mod	Mild	Mod	Mild
	Presence of viral antigen	Yes	Yes	Yes	No	No	No	Yes	Yes	Yes	Yes	Yes	Yes	Yes	Yes	Yes

Abbreviations: WNL, within normal limits; Mod, moderate; and Mkd, marked.

## Discussion

Due to the lack of available vaccines against CCHF, there is an urgent requirement for the development of a modern immunisation approach that can meet international regulatory approval. To date, the MVA-GP vaccine candidate developed against CCHF is the only vaccine that has demonstrated efficacy against lethal CCHFv infection and is attractive for international licensure [11]. It is therefore desirable to obtain more data about this candidate and develop a better understanding of its effects. In this study, MVA-GP vaccinated animals were used as a positive control group, and again demonstrated the 100% efficacy of the vaccine approach. However, due to previous work demonstrating that a prime-boost vaccination with MVA-GP generates both humoral and cellular immunity [11], further work was required to elucidate how each arm of the immune system contributed to the protective effects.

In the present study, it was observed that animals which received both sera and CD3^+^ T-cells from immunised mice exhibited a significant increase in time to death when compared with those animals receiving either sera or CD3^+^ T cells. The finding that both arms of immunity are needed to exert a protective effect has been demonstrated with other viral pathogens. Using a highly pathogenic and cytolytic mouse pathogen, Ectromelia virus, it was demonstrated that antibodies and CD8^+^ T cells were complementary and essential to survival against infection in the natural host [21]. It is envisaged that to protect against CCHF virus, the T cells eliminate intracellular viruses and the humoral response targets extracellular viruses. During vaccine development for another viral haemorrhagic fever virus, Ebola virus, it has also been suggested that vaccines would need to induce protective humoral as well as protective cellular responses to efficiently clear both free virus and virus-infected cells [22]. In this study, although the transfer of both T cells and sera extended the time to death, all animals still met humane clinical endpoints. It is possible that the transfer of the immune mediators were at concentrations too low to provide complete protection. Alternatively, there may have been insufficient continuous stimulus to antigen or a breakdown of cells and antibodies which reduced their protective effects. Due to only a limited volume of cells and antibodies being transferred into the recipient mice, the infection may have overcome the level of adaptive immune response transferred.

To ascertain whether antibodies on their own played a protective role, sera was passively transferred to recipient mice. The protective role of humoral immunity has been demonstrated with other viruses in the *Bunyaviridae* family, including Rift Valley Fever virus (RVFv) [23, 24] and Hantavirus models [25, 26]. In our work antibodies were delivered via the i.p. route, this is an effective route for transfer and other studies have shown that relocation from the peritoneal cavity to the blood occurred within 2 hours [27], however in our study transferred antibodies failed to protect mice from a virus challenge. In contrast passive transfer studies of antibodies generated by a Ross River virus vaccine to naive mice also using the i.p. route where able to transfer protection [15]. In this case 150μl of sera was i.p. transferred to A129, the assumption was made that the serum would be diluted approximately 1:10 in the mouse blood volume [15]. Despite our study delivering a larger volume of 200μl, protection was still not conferred, suggesting that antibodies alone are unable to protect.

Whilst the dogma is that the general aim of vaccine application is the production of neutralising antibodies [6, 9], this may not always hold true. During convalescence from CCHF in humans, the levels of neutralising antibody activity are relatively low [28, 29] and the importance of neutralising antibodies in protection against disease is unknown. Additionally, it has been shown that in CCHFv there is no strict correlation between *in vitro* neutralisation and *in vivo* protection [30]. With a cell-based vaccine against CCHFv that conferred protection, neutralising antibodies were measured and deemed essential for the increased protection of mice [13]; however, the role of other immune responses, including T cell responses, was not assessed. Due to this cell-based vaccine being similar to the existing CCHF vaccine used in Bulgaria which generates T-cell and antibody responses in vaccinated humans [7], it is plausible that both arms of the immune system contribute to the protective effects observed. We have previously shown that not all MVA-GP immunised animals generate antibody responses yet complete protection against lethal CCHFv challenge was observed [17]. Similarly, in this study not all animals showed an antibody response to recombinant CCHF Gn protein yet the MVA-GP immunised group demonstrated complete protection. These observations support the role that antibody alone is unlikely to be the mediator of protection for the MVA-GP vaccine.

To investigate the role of cellular immunity, CD3^+^ T cells from immunised animals were adoptively transferred into recipient mice. The i.p. route was chosen over the standard i.v. route. This was because i.p. injections are more convenient to administer than i.v. injections and they allow the delivery of more cells than can be tolerated via the i.v. route [31]. Additionally, working with A129 mice is technically demanding, as they need to be housed in infection-free flexible film isolators making access to the i.v. route difficult and causing extra distress to mice [32]. Moreover, there is good evidence that immune cells infused via the i.p. route follow a similar course of distribution as those delivered via the i.v. route [33], i.e. migrating to the spleen [31]. Also both i.p. and i.v. transfer have an equilibrated homing time of 24 hours [34].

In the present study, CD3^+^ T cells (containing a crude mixture of both CD4^+^ and CD8^+^ cells) failed to confer protective effects on their own. This is in contrast to passive transfer studies using material derived from different vaccine studies enriched in CD8^+^ T cells, that have transferred protection [22, 35]. For example, using the i.p. route to transfer cytotoxic T-lymphocytes from mice vaccinated with a Venezuelan equine encephalitis virus replicon encoding the Ebola virus nucleoprotein, mice were protected from a lethal Ebola virus challenge even though challenge was performed 4 hours post-cell transfer [22]. Similarly, CD8^+^ T cells delivered i.p. have also been demonstrated to confer protection from West Nile virus [35].

Our results are the first to provide evidence that both the humoral and cellular arms of the immune system are required to exert a protective effect against CCHFv infection after vaccination with a MVA vaccine expressing the CCHFv glycoprotein.

## References

[1] Ergonul O. Crimean-Congo haemorrhagic fever. The Lancet Infectious diseases. 2006;6(4):203–14. Epub 2006/03/24. 10.1016/S1473-3099(06)70435-2 . 16554245 PMC7185836

[2] Bente DA, Forrester NL, Watts DM, McAuley AJ, Whitehouse CA, Bray M. Crimean-Congo hemorrhagic fever: history, epidemiology, pathogenesis, clinical syndrome and genetic diversity. Antiviral research. 2013;100(1):159–89. Epub 2013/08/03. 10.1016/j.antiviral.2013.07.006 . 23906741

[3] Maltezou HC, Andonova L, Andraghetti R, Bouloy M, Ergonul O, Jongejan F, et al Crimean-Congo hemorrhagic fever in Europe: current situation calls for preparedness. Euro surveillance: bulletin Europeen sur les maladies transmissibles = European communicable disease bulletin. 2010;15(10):19504 Epub 2010/04/21. . 20403306

[4] Arda B, Aciduman A, Johnston JC. A randomised controlled trial of ribavirin in Crimean Congo haemorrhagic fever: ethical considerations. Journal of medical ethics. 2012;38(2):117–20. Epub 2011/10/14. 10.1136/medethics-2011-100107 . 21994465

[5] Ascioglu S, Leblebicioglu H, Vahaboglu H, Chan KA. Ribavirin for patients with Crimean-Congo haemorrhagic fever: a systematic review and meta-analysis. The Journal of antimicrobial chemotherapy. 2011;66(6):1215–22. Epub 2011/04/13. 10.1093/jac/dkr136 . 21482564

[6] Papa A, Papadimitriou E, Christova I. The Bulgarian vaccine Crimean-Congo haemorrhagic fever virus strain. Scandinavian journal of infectious diseases. 2011;43(3):225–9. Epub 2010/12/15. 10.3109/00365548.2010.540036 . 21142621

[7] Mousavi-Jazi M, Karlberg H, Papa A, Christova I, Mirazimi A. Healthy individuals' immune response to the Bulgarian Crimean-Congo hemorrhagic fever virus vaccine. Vaccine. 2012;30(44):6225–9. Epub 2012/08/21. 10.1016/j.vaccine.2012.08.003 . 22902680

[8] Spik K, Shurtleff A, McElroy AK, Guttieri MC, Hooper JW, SchmalJohn C. Immunogenicity of combination DNA vaccines for Rift Valley fever virus, tick-borne encephalitis virus, Hantaan virus, and Crimean Congo hemorrhagic fever virus. Vaccine. 2006;24(21):4657–66. Epub 2005/09/22. 10.1016/j.vaccine.2005.08.034 . 16174542

[9] Ghiasi SM, Salmanian AH, Chinikar S, Zakeri S. Mice orally immunized with a transgenic plant expressing the glycoprotein of Crimean-Congo hemorrhagic fever virus. Clinical and vaccine immunology: CVI. 2011;18(12):2031–7. Epub 2011/10/21. 10.1128/CVI.05352-11 22012978 PMC3232705

[10] Bente DA, Alimonti JB, Shieh WJ, Camus G, Stroher U, Zaki S, et al Pathogenesis and immune response of Crimean-Congo hemorrhagic fever virus in a STAT-1 knockout mouse model. Journal of virology. 2010;84(21):11089–100. Epub 2010/08/27. 10.1128/JVI.01383-10 20739514 PMC2953203

[11] Bereczky S, Lindegren G, Karlberg H, Akerstrom S, Klingstrom J, Mirazimi A. Crimean-Congo hemorrhagic fever virus infection is lethal for adult type I interferon receptor-knockout mice. The Journal of general virology. 2010;91(Pt 6):1473–7. Epub 2010/02/19. 10.1099/vir.0.019034-0 . 20164263

[12] Akira S. Functional roles of STAT family proteins: lessons from knockout mice. Stem cells. 1999;17(3):138–46. Epub 1999/05/26. 10.1002/stem.170138 . 10342556

[13] Huang S, Hendriks W, Althage A, Hemmi S, Bluethmann H, Kamijo R, et al Immune response in mice that lack the interferon-gamma receptor. Science. 1993;259(5102):1742–5. Epub 1993/03/19. . 8456301 10.1126/science.8456301

[14] Muller U, Steinhoff U, Reis LF, Hemmi S, Pavlovic J, Zinkernagel RM, et al Functional role of type I and type II interferons in antiviral defense. Science. 1994;264(5167):1918–21. Epub 1994/06/24. . 8009221 10.1126/science.8009221

[15] Holzer GW, Coulibaly S, Aichinger G, Savidis-Dacho H, Mayrhofer J, Brunner S, et al Evaluation of an inactivated Ross River virus vaccine in active and passive mouse immunization models and establishment of a correlate of protection. Vaccine. 2011;29(24):4132–41. Epub 2011/04/12. 10.1016/j.vaccine.2011.03.089 . 21477673

[16] Canakoglu N, Berber E, Tonbak S, Ertek M, Sozdutmaz I, Aktas M, et al Immunization of knock-out alpha/beta interferon receptor mice against high lethal dose of Crimean-Congo hemorrhagic fever virus with a cell culture based vaccine. PLoS neglected tropical diseases. 2015;9(3):e0003579 Epub 2015/03/12. 10.1371/journal.pntd.0003579 25760444 PMC4356576

[17] Buttigieg KR, Dowall SD, Findlay-Wilson S, Miloszewska A, Rayner E, Hewson R, et al A novel vaccine against Crimean-Congo Haemorrhagic Fever protects 100% of animals against lethal challenge in a mouse model. PloS one. 2014;9(3):e91516 Epub 2014/03/14. 10.1371/journal.pone.0091516 24621656 PMC3951450

[18] Chu H, Das SC, Fuchs JF, Suresh M, Weaver SC, Stinchcomb DT, et al Deciphering the protective role of adaptive immunity to CHIKV/IRES a novel candidate vaccine against Chikungunya in the A129 mouse model. Vaccine. 2013;31(33):3353–60. Epub 2013/06/04. 10.1016/j.vaccine.2013.05.059 23727003 PMC3731778

[19] Shimizu S. Routes of Administration In: Hedrich HJ, Bullock G, editors. The Laboratory Mouse: Elsevier; 2004 p. 527–42.

[20] Atkinson B, Chamberlain J, Logue CH, Cook N, Bruce C, Dowall SD, et al Development of a real-time RT-PCR assay for the detection of Crimean-Congo hemorrhagic fever virus. Vector borne and zoonotic diseases. 2012;12(9):786–93. Epub 2012/01/06. 10.1089/vbz.2011.0770 . 22217175

[21] Fang M, Sigal LJ. Antibodies and CD8+ T cells are complementary and essential for natural resistance to a highly lethal cytopathic virus. Journal of immunology. 2005;175(10):6829–36. Epub 2005/11/08. . 16272340 10.4049/jimmunol.175.10.6829

[22] Wilson JA, Hart MK. Protection from Ebola virus mediated by cytotoxic T lymphocytes specific for the viral nucleoprotein. Journal of virology. 2001;75(6):2660–4. Epub 2001/02/27. 10.1128/JVI.75.6.2660-2664.2001 11222689 PMC115890

[23] Bhardwaj N, Heise MT, Ross TM. Vaccination with DNA plasmids expressing Gn coupled to C3d or alphavirus replicons expressing gn protects mice against Rift Valley fever virus. PLoS neglected tropical diseases. 2010;4(6):e725 Epub 2010/06/29. 10.1371/journal.pntd.0000725 20582312 PMC2889828

[24] Schmaljohn CS, Parker MD, Ennis WH, Dalrymple JM, Collett MS, Suzich JA, et al Baculovirus expression of the M genome segment of Rift Valley fever virus and examination of antigenic and immunogenic properties of the expressed proteins. Virology. 1989;170(1):184–92. Epub 1989/05/01. . 2655274 10.1016/0042-6822(89)90365-6

[25] Custer DM, Thompson E, Schmaljohn CS, Ksiazek TG, Hooper JW. Active and passive vaccination against hantavirus pulmonary syndrome with Andes virus M genome segment-based DNA vaccine. Journal of virology. 2003;77(18):9894–905. Epub 2003/08/28. 12941899 10.1128/JVI.77.18.9894-9905.2003 PMC224585

[26] Hooper JW, Ferro AM, Wahl-Jensen V. Immune serum produced by DNA vaccination protects hamsters against lethal respiratory challenge with Andes virus. Journal of virology. 2008;82(3):1332–8. Epub 2007/11/23. 10.1128/JVI.01822-07 18032485 PMC2224461

[27] Mattes MJ. Biodistribution of antibodies after intraperitoneal or intravenous injection and effect of carbohydrate modifications. Journal of the National Cancer Institute. 1987;79(4):855–63. Epub 1987/10/01. . 2443739

[28] Keshtkar-Jahromi M, Kuhn JH, Christova I, Bradfute SB, Jahrling PB, Bavari S. Crimean-Congo hemorrhagic fever: current and future prospects of vaccines and therapies. Antiviral research. 2011;90(2):85–92. Epub 2011/03/03. 10.1016/j.antiviral.2011.02.010 . 21362441

[29] Shepherd AJ, Swanepoel R, Leman PA. Antibody response in Crimean-Congo hemorrhagic fever. Reviews of infectious diseases. 1989;11 Suppl 4:S801–6. Epub 1989/05/01. . 2501854 10.1093/clinids/11.supplement_4.s801

[30] Bertolotti-Ciarlet A, Smith J, Strecker K, Paragas J, Altamura LA, McFalls JM, et al Cellular localization and antigenic characterization of crimean-congo hemorrhagic fever virus glycoproteins. Journal of virology. 2005;79(10):6152–61. Epub 2005/04/29. 10.1128/JVI.79.10.6152-6161.2005 15858000 PMC1091677

[31] Petersen CC, Petersen MS, Agger R, Hokland ME. Accumulation in tumor tissue of adoptively transferred T cells: A comparison between intravenous and intraperitoneal injection. Journal of immunotherapy. 2006;29(3):241–9. Epub 2006/05/16. 10.1097/01.cji.0000203078.97493.c3 . 16699367

[32] Yardeni T, Eckhaus M, Morris HD, Huizing M, Hoogstraten-Miller S. Retro-orbital injections in mice. Lab animal. 2011;40(5):155–60. Epub 2011/04/22. 10.1038/laban0511-155 21508954 PMC3158461

[33] Du X, Jin R, Ning N, Li L, Wang Q, Liang W, et al In vivo distribution and antitumor effect of infused immune cells in a gastric cancer model. Oncology reports. 2012;28(5):1743–9. Epub 2012/09/06. 10.3892/or.2012.2013 . 22948809

[34] Matheu MP, Cahalan MD, Parker I. General approach to adoptive transfer and cell labeling for immunoimaging. Cold Spring Harbor protocols. 2011;2011(2):pdb prot5565. Epub 2011/02/03. 10.1101/pdb.prot5565 . 21285265

[35] Shrestha B, Samuel MA, Diamond MS. CD8+ T cells require perforin to clear West Nile virus from infected neurons. Journal of virology. 2006;80(1):119–29. Epub 2005/12/15. 10.1128/JVI.80.1.119-129.2006 16352536 PMC1317548

